# Enhanced Recovery after Elective Open Surgical Repair of Abdominal Aortic Aneurysm: A Complementary Overview through a Pooled Analysis of Proportions from Case Series Studies

**DOI:** 10.1371/journal.pone.0098006

**Published:** 2014-06-02

**Authors:** Sanderland J. T. Gurgel, Regina El Dib, Paulo do Nascimento

**Affiliations:** 1 Department of Anesthesiology, Faculdade de Medicina de Botucatu, UNESP, Univ Estadual Paulista, Brazil; and UNINGÁ University, Maringá, Paraná, Brazil; 2 Department of Anesthesiology, Faculdade de Medicina de Botucatu, UNESP, Univ Estadual Paulista, Brazil; and McMaster Institute of Urology, McMaster University, Canada; 3 Department of Anesthesiology, Faculdade de Medicina de Botucatu, UNESP, Univ Estadual Paulista, Brazil; San Raffaele Scientific Institute, Italy

## Abstract

**Objectives:**

To evaluate the efficacy and safety of enhanced recovery after surgery (ERAS) programs in elective open surgical repair (OSR) of abdominal aortic aneurysm (AAA).

**Background:**

Open surgical repair of AAA is associated with high morbidity and mortality, prolonged hospital stay and high costs. ERAS programs contribute to the optimization of treatment by reducing hospital stay and improving clinical outcomes.

**Methods:**

A review of PubMed, EMBASE and LILACS databases was conducted. As only one randomized controlled trial was found, a pooled analysis of proportions from case series was conducted, considering it a complementary overview of the topic. Inclusion criteria were case series with more than five cases reported, adult patients who underwent an elective OSR of AAA and use of an ERAS program. ERAS was compared to conventional perioperative care. The pooled proportion and the confidence interval (CI) are shown for each outcome. The overlap of the CI suggests similar effect of the interventions studied.

**Results:**

Thirteen case series studies with ERAS involving 1,250 patients were compared to six case series with conventional care with a total of 1,429 patients. The pooled, respective proportions for ERAS and conventional care were: mortality, 1.51% [95% CI: 0.0091, 0.0226] and 3.0% [95% CI 0.0183, 0.0445]; and incidence of complications, 3.82% [95% CI 0.0259, 0.0528] and 4.0% [95% CI 0.03, 0.05].

**Conclusion:**

This review shows that ERAS and conventional care therapies have similar mortality and complication rates in OSR of AAA.

## Introduction

Elective open surgical repair of the abdominal aortic aneurysm (AAA) has been the most effective treatment to prevent its rupture when considering anatomically more complex aneurysms with a diameter greater than 5.5 cm. On the other hand, currently, endovascular aortic aneurysm repair is progressively replacing open surgical repair for the treatment of infrarenal AAA and now accounts for more than half of all AAA repairs [1]. The open surgical repair is a major procedure with extended surgical incisions that involves opening the abdomen for several hours and aortic clamping for graft interposition, requiring prolonged anesthetic/surgery time and, sometimes, blood transfusions. As a result, there may be an increased incidence of cardiorespiratory complications and prolonged intensive care unit length of stay, and a long period before returning to routine activities [2].

Recently, a more systematic perioperative approach targeting the reduction of costs and the improvement of outcomes, consisting of multimodal strategies has replaced conventional perioperative care. These new strategies involve a combination of various different treatments, i.e., a reduced fasting time, less invasive surgical procedures, a better strategy for postoperative pain control, use of short-acting anesthetics, ileus control and the rational use of invasive monitoring and intensive care treatment [3]. These strategies have been adopted mainly for colorectal surgeries [4], but also for nephrectomy [5], thyroidectomy [6] and hip arthroplasty [7] and are called enhanced recovery after surgery (ERAS) programs.

Due to a lack of clinical trials on comparing the use of ERAS with the conventional perioperative care for elective open surgical repair of AAA, a pooled analysis of proportions from case series studies [8], [9] was performed to enable comparison of the available scientific evidence. This study sought to improve current practice and outline new potential directions for future research and the development of randomized controlled trials [10]–[12].

The rationale for this study is that a perioperative strategy to reduce costs and to improve short-term outcomes seems to be a very sensible choice when an elective open surgical repair of an AAA is performed.

## Methods

A review of clinical case series with pooled analysis of proportions of patients in elective open surgical repair of AAA with the use of an ERAS program was performed. The method used in this study to perform the pooled analysis of proportions from case series was previously described in detail by El Dib et al., 2013 [13]. A database search with no language restriction was made on the following sources (last update July 2013): Pubmed (1966–2013), EMBASE (1980–2013) and LILACS (1982–2013). The search aimed to identify all case series concerning the use of ERAS programs or perioperative conventional care. The following comprehensive search strategy was used: ((ERAS OR enhanced recovery after surgery OR fast track OR multimodal rehabilitation OR hospital length of stay) AND (abdominal aortic aneurysm OR aortic aneurysm OR aortic surgery OR aortic repair)). The search strategy was adapted for each database to achieve more sensitivity.

The reference lists of all relevant papers and published review articles were searched. The online trials registers www.clinicaltrials.gov and www.controlled-trials.com for published and unpublished studies were also searched. After removing duplicates, the articles were selected to evaluate the title and abstract. Two reviewers (SJTG and PNJ) independently evaluated the titles and abstracts, and in the case of disagreement, a third reviewer (RED) was consulted for resolution. In case of a non-English written manuscript, a language expert was consulted for translation.

The following inclusion criteria were used: (i) case series studies with more than five reported cases with at least a 30-day follow-up; (ii) patients older than 18 years who underwent an elective open surgical repair of AAA; (iii) use of an ERAS program, defined as the use of at least four out of a total of 15 previously established items aiming to reduce hospital length of stay and hospital costs, or perioperative conventional care [14]; and (iv) a reported 30-day mortality, as the primary outcome.

Secondary outcome was the rate of complications or morbidity, defined as the absolute number of patients with at least one postoperative complication (acute myocardial infarction, characterized by electrocardiographic elevation of the ST segment above 0.1 mV in 2 leads, or biochemical elevation of CK-MB mass or troponin enzymes; renal failure, characterized by the need for renal replacement therapy; and stroke, defined as the change in consciousness or motor level developed postoperatively or proof of injury by brain imaging.

Due to the difficulty in defining conventional perioperative care, and although the comparison between endovascular repair and elective open surgical repair was not the focus of this systematic review, it was decided to use results for patients undergoing open surgical repair of AAA in studies where there was randomization and comparison with endovascular repair. This approach verified that the perioperative care provided to patients who underwent open surgical repair had not employed an ERAS strategy, but rather represented conventional care based on the best-available medical practice. Six randomized controlled trials were published between 2004 and 2011 comparing endovascular repair with open surgical repair. These data were summarized in a recently published meta-analysis [15].

If there were more than one published study on the same group of patients, the articles were analyzed to verify whether they reported different outcomes or not. If they presented the same outcome data, the data were extracted from the most recent or most complete article. The mean age and follow-up calculated in this study were based on the mean age and follow-up of each case series included in this review.

### Statistical analysis

The morbidity and mortality rates for the case series studies were treated as dichotomous variables with their respective confidence intervals (CI) of 95%. Because of clear differences between the included studies and several uncontrollable variables, a random-effects model was used to perform the pooled analysis of proportions [16]. The program used to perform the meta-analysis was the StatsDirect (StatsDirect Ltd, UK) [8].

Forest plot charts were presented to summarize the data. Each horizontal line on the graph represents a case series included in the meta-analysis. The estimated effect is marked with a solid black square, and the size of the square represents the weight of the corresponding study plotted in the meta-analysis. The combined total estimate is marked with an unfilled diamond at the bottom of the forest plot. Combined proportionality and the 95% CIs are presented. The presence of an overlap of the confidence intervals from the two interventions, ERAS and conventional perioperative care, suggests similar effect of the interventions on the outcome. On the other hand, non-overlapping CIs suggest different effects from the interventions studied.

## Results

**Figure 1:**
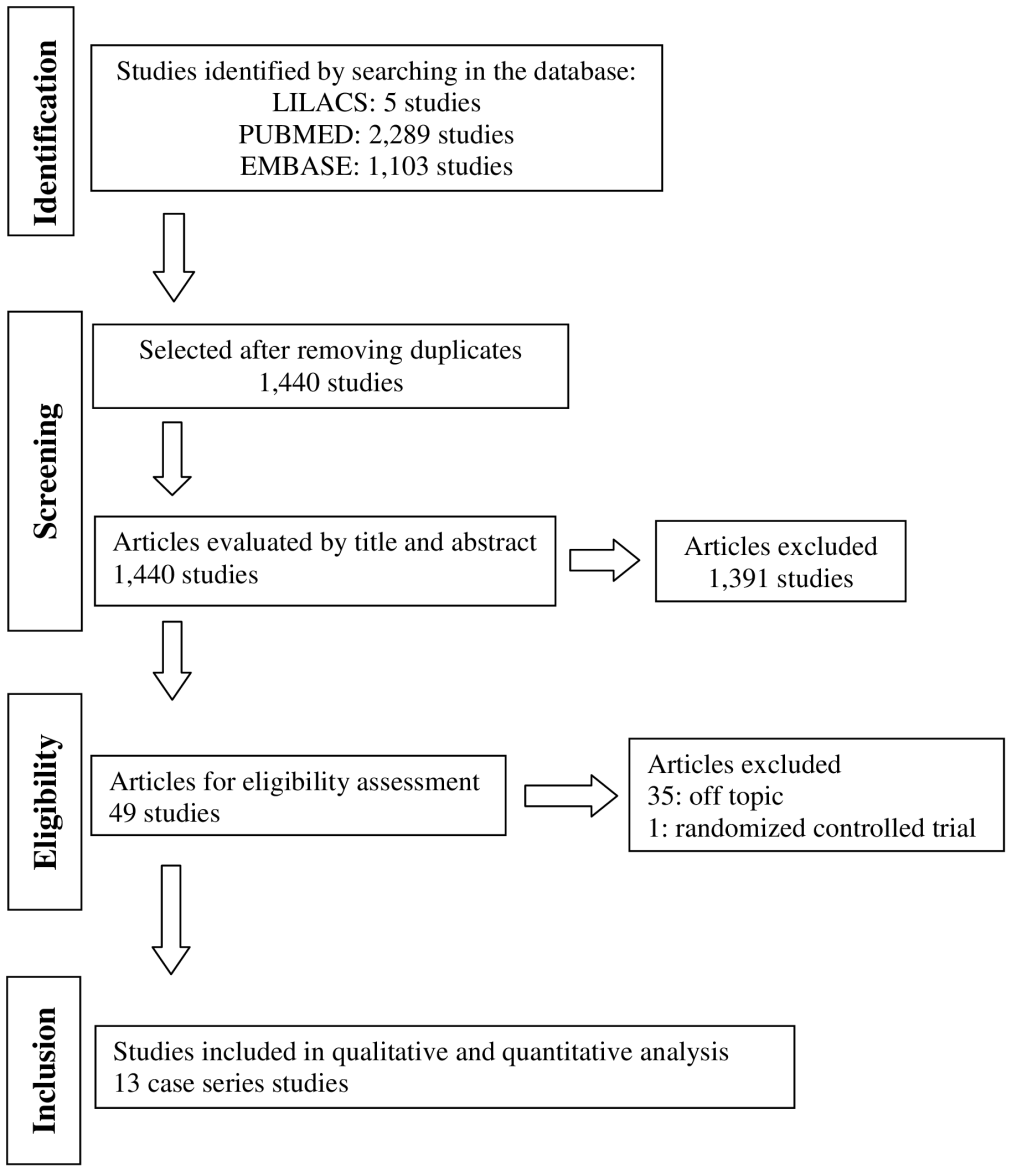
Flow chart showing the number of abstracts and articles identified and evaluated during the review process.

The search was conducted until July 2013 and identified 1,440 titles. After screening by title and then abstract, we obtained full paper copies of 49 studies on ERAS and conventional perioperative care that were potentially eligible for inclusion in the review. However, most of these studies were either retrospective, animal studies, reviews, or did not evaluate a relevant clinical outcome. A total of 13 case series studies with 1,250 patients met all inclusion criteria and were selected for the pooled analysis of proportions (Figure 1).

The demographic characteristics and perioperative data are presented in Tables 1 and 2, respectively, for patients in which ERAS was performed. Data from the control group involving 1,429 patients treated with conventional perioperative care are shown in Table 3. The year of publication of the ERAS case series ranged from 1999 to 2011. The studies were distributed across North America, Europe and Asia. The mean patient age was 70 years, 84% were male, 62% were smokers, 63% had hypertension, 42% had prior heart disease and 11% were diabetic. The domains associated with ERAS programs are described in Table 4.

**Table 1:** Demographic data of case series studies of ERAS programs included in this review.

Study	Year	Country	n	Age, years mean (range)	Gender, male/female n/n	Smokers n (%)	Hypertension n (%)	Cardiac disease n (%)	Diabetes n (%)
Abularrage et al. [28]	2005	USA	30	68 (66–70)	25/5	23 (77%)	24 (80%)	14 (47%)	4 (13%)
Brustia et al. [29]	2007	Italy	323	70 (50–87)	294/29	_	_	_	_
Callaghan et al. [30]	2005	UK	178	72 (66–77)	156/22	57 (38%)	73 (48%)	45 (29%)	12 (8%)
Chang et al. [31]	2003	USA	240	69 (60–80)	171/69	208 (87%)	167 (70%)	113 (47%)	29 (12%)
Hafez et al. [32]	2011	UK	83	73 (61–87)	77/6	11 (13%)	48 (58%)	47 (57%)	13 (16%)
Ko et al. [33]	2004	China	10	73 (63–89)	7/3	_	8 (80%)	_	2 (20%)
Löhr et al. [34]	2008	Germany	35	71 (59–83)	31/4	_	_	_	_
Moniaci et al. [35]	2011	Italy	94	71 (63–79)	84/10	80 (85%)	84 (89%)	–	9 (10%)
Mukherjee et al [36]	2008	USA	30	67 (60–88)	25/5	_	_	_	_
Murphy et al. [37]	2007	UK	30	73 (50–89)	26/4	–	–	–	–
Podore et al. [38]	1999	USA	50	64 (40–88)	34/16	37 (74%)	26 (52%)	24 (48%)	3 (6%)
Renghi et al. [39]	2001	Italy	58	66 (55–75)	–	–	–	–	–
Rigberg et al. [40]	2004	USA	89	73	71/18	58 (65%)	58 (65%)	37 (42%)	13 (15%)
Total values, n (range or %)			1,250	70 (40–89)	1001 (84%)/191 (16%)	474 (62%)	488 (63%)	280 (42%)	85 (11%)

**Table 2:** Perioperative data from the case series studies included in review.

Study	Aortic diameter, cm (range)	Surgery length, min mean	Extubation site	ICU* length of stay, days mean	Morbidity rate† n (%)	Acute myocardial infarction n (%)	Renal failure n (%)	Stroke n (%)	Mortality rate n (%)	Hospital length of stay, days mean (range)
Abularrage et al. [28]	N/A‡	157	LMA§, OR∥	N/A	4 (13)	0	1 (3)	0	1 (3)	3 (1–7)
Brustia et al. [29]	N/A	175	OR	1	16 (5)	6 (2%)	10 (3)	0	8 (2%)	3 (1–21)
Callaghan et al. [30]	N/A	N/A	OR	1	85 (56)	N/A	10 (7)	0	2 (1)	12 (2–28)
Chang et al. [31]	5.9 (5.1–10)	150	OR	1	44 (18)	3 (1%)	2 (1%)	0	1 (0.5)	8 (4–28)
Hafez et al. [32]	6.8 (5.5–9)	N/A	ICU	1	18 (22%)	2 (2%)	2 (2%)	1 (1%)	2 (2%)	4 (2–88)
Ko et al. [33]	N/A	160	OR	N/A	0	0	0	0	0	5 (3–8)
Löhr et al. [34]	6.1 (5–8.5)	175	N/A	1	5 (15%)	1 (3%)	1 (3%)	0	0	N/A
Moniaci et al. [35]	N/A	N/A	OR	1	14 (15%)	1 (1%)	1 (1%)	0	2 (2%)	4 (2–17)
Mukherjee et al [36]	6.5 (5.5–8.4)	140	OR	1	2 (7%)	0	2 (7%)	0	1 (3%)	3 (3–7)
Murphy et al. [37]	N/A	N/A	PACU¶	N/A	18 (54%)	N/A	N/A	N/A	0	5 (2–12)
Podore et al [38]	6.1 (6.3–5.9)	216	OR	1	6 (12%)	1 (2%)	N/A	0	0	3 (2–8)
Renghi et al. [39]	5.5 (4.3–6.8)	151	LMA, OR	N/A	1 (2%)	0	0	0	0	7.6 (5–10)
Rigberg et al. [40]	N/A	N/A	N/A	N/A	7 (8%)	N/A	N/A	N/A	0	5 (2–11)
Total values n (range, mean or %)	6.15 (4.3–10)	165.5		1	220 (18%)	14 (1%)	29 (2%)	1 (0.1%)	17 (1%)	5.21 (1–88)

*ICU  =  intensive care unit;

† Morbidity rate  =  absolute number of patients with at least one complication described in the included article;

‡ N/A  =  not applicable;

§ LMA  =  laryngeal mask airway;

∥ OR  =  operating room;

¶ PACU  =  post anesthetic care unit.

**Table 3:** Perioperative data and demographics of patients submitted to elective open surgical repair of AAA from comparative studies with endovascular repair of AAA and submitted to conventional perioperative care.

Study	n	Age, years mean	Gender (male/female) n/n	Smokers n (%)	Hypertension n (%)	Cardiac disease n (%)	Diabetes n (%)	Aortic diameter, cm mean	Hospital length of stay, days mean	ICU∥ length of stay, days mean	Mortality rate n
ACE* [41]	149	70	146/3	146 (98%)	95 (64%)	65 (44%)	29 (20%)	5.56	10.4	N/A¶	1
DREAM† [42]–[44]	178	70	161/17	161 (90%)	97 (55%)	83 (47%)	17 (10%)	6	10	1	8
EVAR-1‡ [45]–[47]	626	74	570/56	580 (91%)	N/A	261 (42%)	68 (11%)	6.5	N/A	N/A	24
OVER§ [48]	437	70	435/2	413 (94%)	330 (75%)	185 (42%)	100 (23%)	5.7	7	4	10
Soulez et al., 2005 [49]	20	71	20/0	16 (80%)	10 (50%)	14 (60%)	5 (25%)	5.1	11.5	1	0
Lottman et al., 2004 [50]	19	69	16/3	N/A	N/A	N/A	N/A	5.6	11	1	1
Total values n (range, mean or %)	1,429	70.6	1,348 (94%)/81 (6%)	1,316 (93%)	532 (68%)	608 (43%)	219 (15%)	5.7	10	1.7	44 (3%)

^*^ACE  =  Aneurysme de l'aorte abdominale; Chirurgie versus Endoprothese;

† DREAM  =  Dutch randomized Endovascular aneurysm management;

‡ EVAR-1  =  Endovascular aneurysm repair;

§ OVER  =  Open versus endovascular repair;

∥ ICU  =  intensive care unit;

¶ N/A  =  not applicable.

**Table 4:** Description of the domains of the ERAS program for each included study.

Studies	1	2	3	4	5	6	7	8	9	10	11	12	13	14	15
Abularrage et al. [28]					X		X	X	X			X	X	X	X
Brustia et al. [29]	X			X		X	X	X	X	X		X	X	X	X
Callaghan et al. [30]	X						X			X		X			
Chang et al. [31]	X						X		X			X		X	X
Hafez et al. [32]	X						X		X			X	X	X	X
Ko et al. [33]							X	X				X	X	X	X
Löhr et al. [34]			X		X		X	X	X			X	X	X	X
Moniaci et al. [35]	X			X			X	X	X	X		X	X	X	X
Mukherjee et al. [36]	X				X		X	X	X			X	X	X	X
Murphy et al. [37]	X					X	X		X			X		X	X
Podore et al. [38]								X				X	X	X	X
Renghi et al. [39]	X			X		X	X	X		X		X		X	X
Rigberg et al. [40]							X	X	X			X	X	X	X

1. Oral and written preadmission counseling and information.

2. Lack of routine preoperative bowel preparation.

3. Reduced fasting time plus carbohydrate loading.

4. Short-acting preanesthetic medication.

5. Prophylaxis against thromboembolism.

6. Antimicrobial prophylaxis.

7. Short-acting opioids/Epidural analgesia.

8. Prevention and treatment of postoperative nausea and vomiting.

9. Less invasive surgical incisions.

10. Prevention of intraoperative hypothermia.

11. Standardization of perioperative fluid management.

12. Judicious use of drains and catheters.

13. Prevention of postoperative ileus.

14. Postoperative nutritional care.

15. Early mobilization.

**Figure 2:**
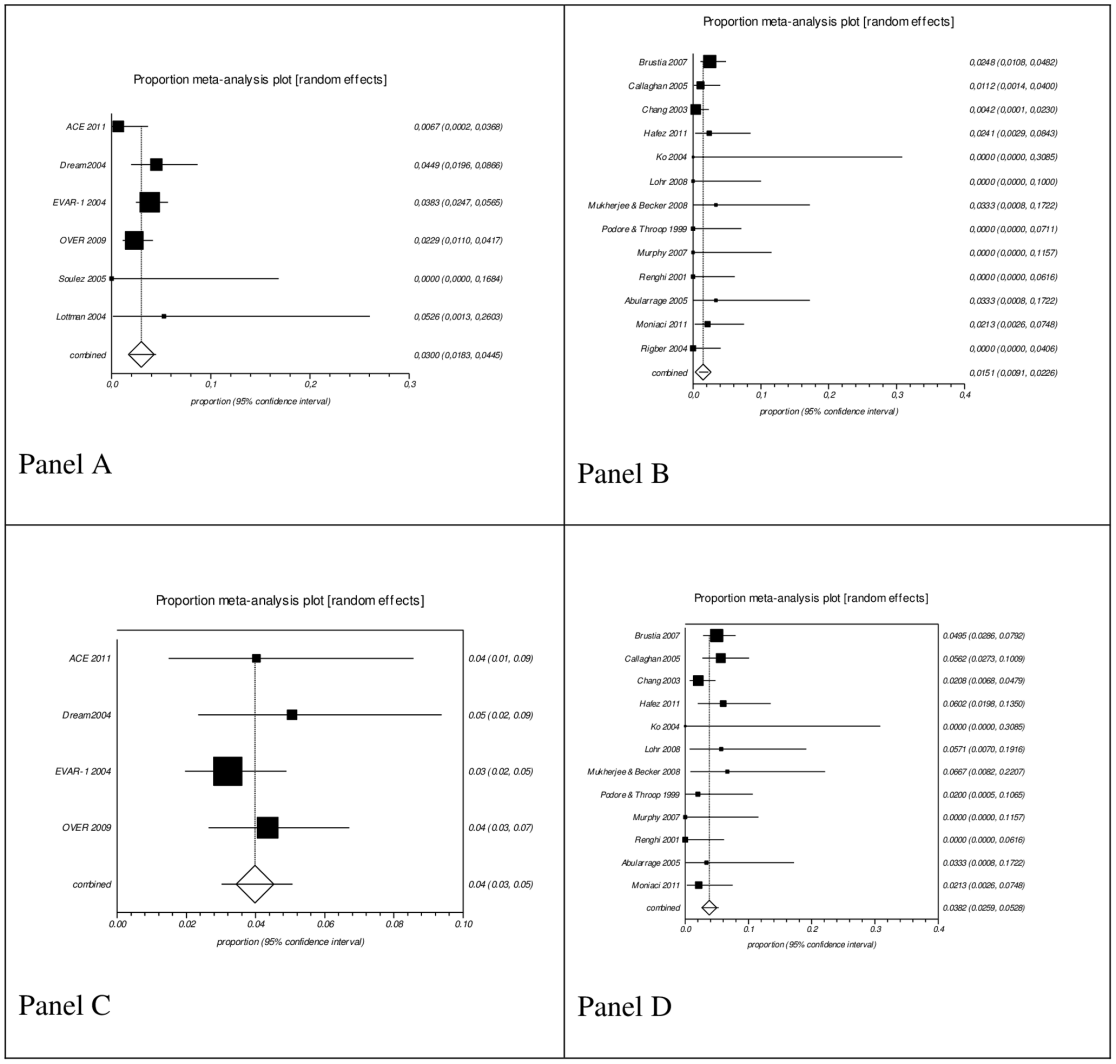
Pooled analysis of proportions from case series. Panel A: Conventional care series mortality; Panel B: ERAS series mortality; Panel C: Conventional care series morbidity; Panel D: ERAS series morbidity. Morbidity results are shown as the absolute number of patients with at least one complication, including acute myocardial infarction, renal failure and stroke. No effect differences were seen due to the overlap of the 95% confidence intervals.

The pooled proportion in ERAS group from 13 case series studies with a total of 1,250 patients and in conventional perioperative care from six studies with a total of 1,429 patients were, respectively, and for each outcome (Figure 2): mortality, 1.51% [95% CI 0.0091, 0.0226] and 3% [95% CI 0.0183, 0.0445]; and incidence of complications (morbidity), 3.82% [95% CI 0.0259, 0.0528] and 4% [95% CI 0.03, 0.05].

**Figure 3:**
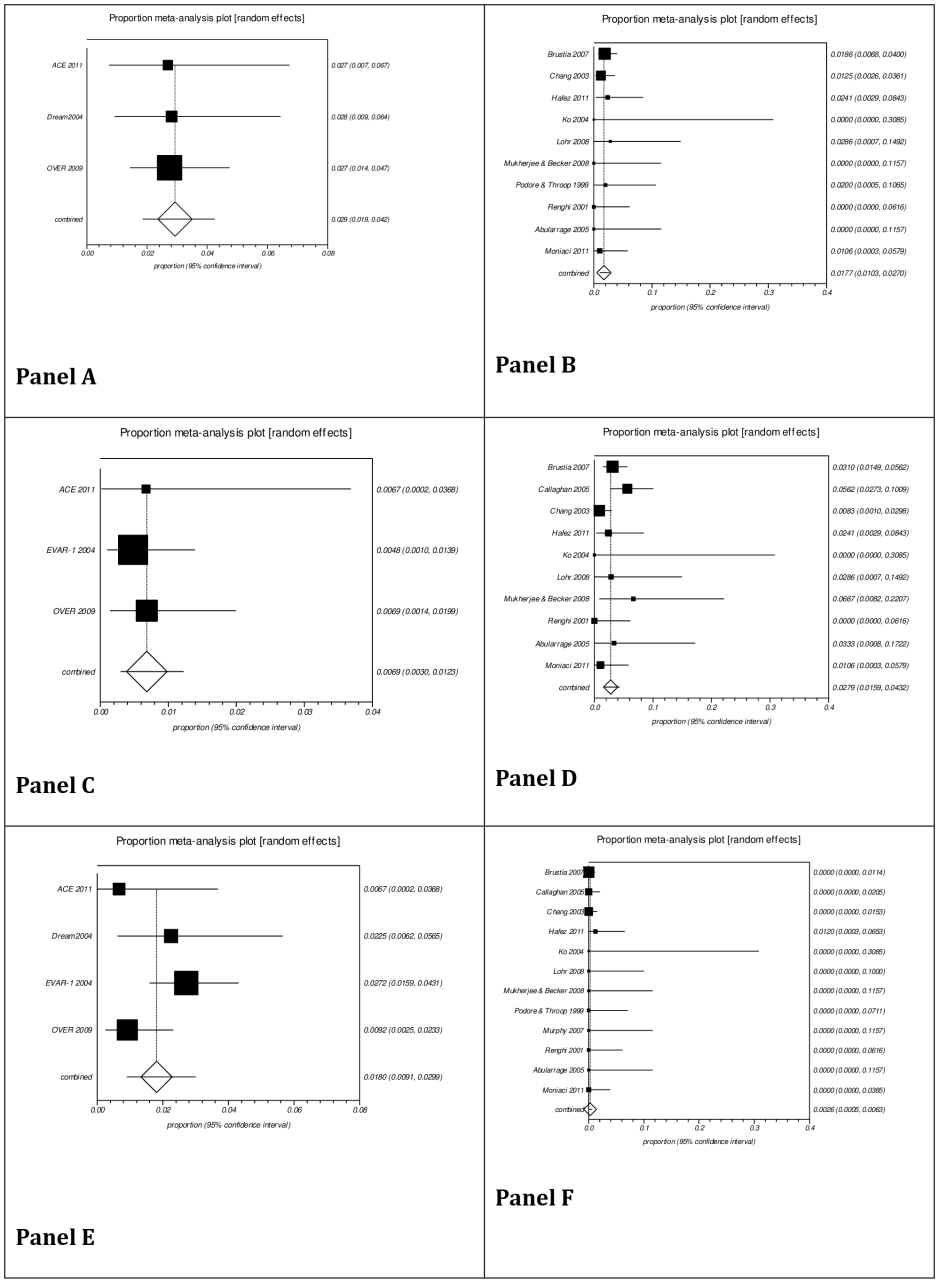
Pooled analysis of proportions from case series related to stratified morbidity. Panel A: Acute myocardial infarction, Conventional care series; Panel B: Acute myocardial infarction, ERAS case series; Panel C: Renal failure, Conventional care series; Panel D: Renal failure, ERAS case series; Panel E: Stroke, Conventional care series; Panel F: Stroke, ERAS case series. An effect difference was seen due to the non-overlap of the 95% confidence intervals in relation to renal failure (favoring conventional care) and stroke (favoring ERAS). No effect difference was seen due to the overlap of the 95% confidence interval considering incidence of acute myocardial infarction.

Subgroup analysis for incidence of complications is presented in Figure 3. The pooled proportion in ERAS group from 13 case series studies with a total of 1,250 patients and in conventional perioperative care from six studies with a total of 1,429 patients were, respectively: acute myocardial infarction, 1.77% [95% CI 0.0103, 0.0270] and 2.9% [95% CI 0.019, 0.042]; renal failure, requiring renal replacement therapy, 2.79% [95% CI 0.0159, 0.0432] and 0.69% [95% CI 0.0030, 0.0123]; stroke, 0.26% [95% CI 0.0005, 0.0063] and 1.8% [95% CI 0.0091, 0.0299].

## Discussion

Systematic reviews of randomized controlled trials on therapeutic interventions are the best study design for decision-making in clinical practice. However, they often provide inconsistent evidence or uncertain conclusions, especially when there is a lack of randomized controlled trials [17], [18]. For this reason, other strategies to deal with the absence of randomized controlled trials have become necessary.

In our search, just one randomized controlled trial comparing ERAS in elective open surgical repair of AAA to conventional perioperative care was identified [19]. Due to the lack of randomized controlled trials in this topic, we performed a pooled analysis of proportions from case series studies [8], [9], [13] as a complementary overview of this topic. The pooled analysis of proportions from case series provide support for clinical practice until high-quality primary studies are conducted, although clinical and methodological heterogeneity is observed due to the nature of case series studies [8], [9], [13].

This review identified 13 case series studies in which ERAS was used with the aim of accelerating postoperative recovery. A total of 1,250 individuals underwent ERAS programs, and their comorbidities correspond to those reported in the literature [20]. The difficulty in obtaining conventional perioperative care case series led our group decide to use the open surgical repair branch of studies randomized for comparison with endovascular aortic aneurysm repair. These data were summarized in a recently published meta-analysis [15], and their results with a 30-day follow-up were used in the present study. These patients' demographics were comparable to those of the ERAS case series (Tables 1 and 3).

The fact that the case series were reported in recent publications (1999 to 2011) along with their geographical distribution (five studies in North America, seven in Europe and one in Asia) demonstrates a global concern regarding the need to change the perioperative approach in AAA. Therefore, the study of case series due to the lack of randomized controlled trials in this field seems to be appropriate and allows a more comprehensive analysis of the collected available evidence.

The mean number of ERAS items for each of the 13 case series studies was eight, ranging from four to 13. Although the use of more items was suggested, only those formally described in the methodology of each study were used. The multimodal approach for enhanced recovery after surgery targets the reduction of overall stress, i.e., social, psychological and physical during the perioperative period, and reduces the time required for recovery [3], [21], [22].

AAA rupture is a catastrophic event with mortality rates reaching 80% [23]. The most commonly used predictor of rupture is the maximum diameter of the aneurysm (above 5.5 cm) [24]. Elective surgery together with a program to enhance recovery seems to be very rational as a better result at a lower cost would favor patients and health care system. The results of the pooled proportions showed a lower combined proportion in mortality rate in favor of ERAS (1.51%) compared to conventional perioperative care (3%). However, due to the overlap of their respective 95% confidence intervals, probably the effect of ERAS and conventional perioperative care on mortality rate is not different. The same observation is made for the overall incidence of complications. No effect difference was seen between ERAS (3.82%) and conventional perioperative care (4%). When analyzing the perioperative complications, it was verified that there was no effect difference of the interventions on the incidence of acute myocardial infarction due to the overlap of the 95% confidence interval. On the other hand, an effect difference due to the non-overlap of the confidence intervals favored conventional perioperative care on renal failure incidence, but favored the ERAS group on stroke incidence.

The StatsDirect software used in the present study to perform the pooled analysis of proportions does not plot continuous variables and, for this reason, we were not able to analyze the effects of ERAS and conventional care on the hospital length of stay. An overview of this variable is shown on Tables 2 and 3.

Major surgeries such as elective open AAA repair are performed worldwide each year and account for a high utilization of financial resources, especially in developed countries [25]. The demand for the highest quality of health care, combined with the need for the rational use of both public and private resources, have become a challenge to the professionals to ensure quality and patient safety at the lowest cost [26]. As a result, ERAS programs tend to gradually replace the so-called conventional perioperative care, providing a more rational treatment.

Potential biases and high heterogeneity most likely occur in nonrandomized studies, and their results should be interpreted with caution. Particularly in our analysis, one potential source of bias is that ERAS series are reports of consecutive cases done by different groups of doctors, with a high chance of heterogeneity among individuals. On the other hand, the conventional perioperative care group (control group) was selected from randomized controlled trials and probably less heterogeneity in these subjects has occurred. When comparing very different and heterogeneous subjects, conclusions have to be made cautiously and the readers must be aware of this issue. Different subjects not randomly selected and not blinded to treatment may respond very differently to the same intervention, decreasing the external validity of the results. Then, the possibility of high heterogeneity in our study may have influenced the results. Due to the nature of the procedure, i.e., AAA repair, the patients tend to have the same clinical profile (age, gender, comorbidities) and probably similar diameter of the aorta, one of the criteria for surgical selection. We were careful when analyzing clinical characteristics of patients from both groups and verified that they had similar profile, tending to reduce the potential heterogeneity. We also considered the number of participants in both groups adequate for the comparison we proposed to do, understanding that the number of subject does not mean quality and homogeneity.

We are aware that this methodology, i.e., pooled analysis of proportions from case series and the estimate of the interventions results according to the overlap of the 95% confidence interval is not strong. Nonetheless, it is a strategy to analyze available data from case series. Some other reasons encourage performing a pooled analysis of proportions from case series studies [27]: support further randomized controlled trials evaluating the potential weaknesses of the topic under discussion, including the identification of relevant subgroups; provide evidence of either beneficial or harmful effects of an intervention that cannot be randomized; and provide evidence of either beneficial or harmful effects that cannot adequately be studied in randomized controlled trials, such as rare conditions or outcomes, and those requiring long follow-ups.

The pooled analysis of proportions from case series is an alternative approach for clinical research until well-conducted clinical trials are conducted. The health care professionals shoud weigh the benefits/risks profile of this approach and also take into consideration the patients' values and preferences.

## Conclusions

The present pooled analysis of proportions from case series shows that ERAS programs and conventional perioperative care therapies are similar considering 30-day mortality and overall complication rates in elective open surgical repair of AAA. However, further and well-conducted randomized controlled trials are necessary to determine the real value of ERAS in elective open AAA surgery.

## Supporting Information

Checklist S1 **PRISMA Checklist.**
(DOC)
